# Supplementary material for: The CNK–HYP scaffolding complex promotes RAF activation by enhancing KSR–MEK interaction
Source: Nat Struct Mol Biol. 2024 Feb 22;31(7):1028–38. doi: 10.1038/s41594-024-01233-6 (PMC11257983; doi:10.1038/s41594-024-01233-6)
Supplement: Supplementary file 1 — Supplementary Figs. 1–4. [file 41594_2024_1233_MOESM1_ESM.pdf]

nature structural & molecular biology

Article

https://doi.org/10.1038/s41594-024-01233-6

The CNK–HYP scaffolding complex promotes RAF activation by enhancing KSR–MEK interaction

In the format provided by the authors and unedited

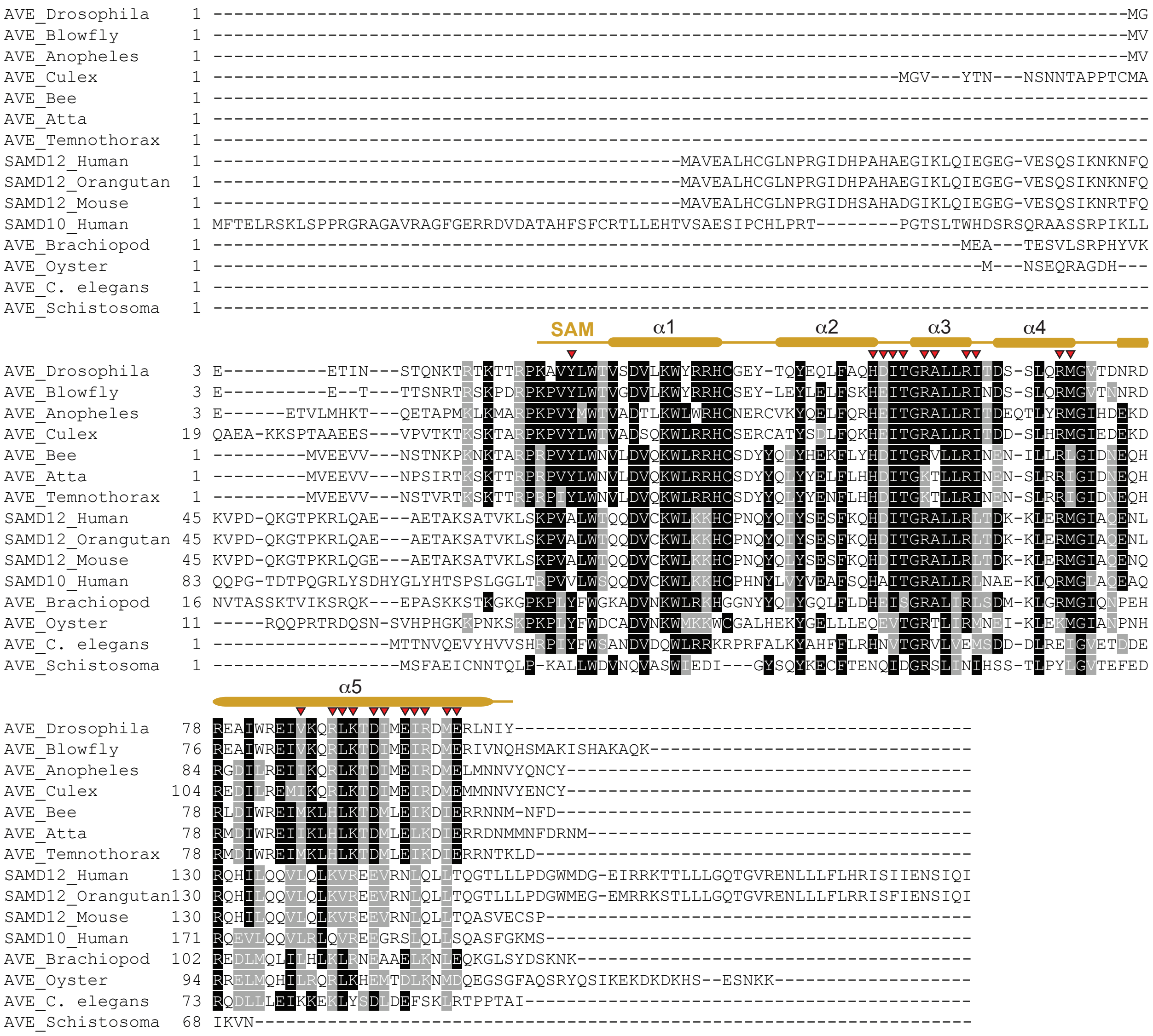

Supplementary Fig. 1 | Structure-based sequence alignment of HYP homologs.

Sequence alignment of Hyphen (Aveugle) homologs from 14 metazoan species. Secondary structure elements of the SAM domain are indicated on top of the alignment. Red triangles indicate residues that are conserved at the binding interfaces with CNK. Species correspond to Drosophila melanogaster (Drosophila), Lucilia cuprina (Blowfly), Anopheles gambiae (Anopheles), Culex quinquefasciatus (Culex), Apis cerana (APICE), Atta cephalotes (Atta), Temnothorax longispinosus (Temnothorax), Homo sapiens (Human), Pongo abelii (Orangutan), Mus musculus (Mouse), Lingula unguis (Brachiopod), Crassostrea gigas (Oyster), Caenorhabditis elegans (C. elegans), Schistosoma mansoni (Schistosoma).

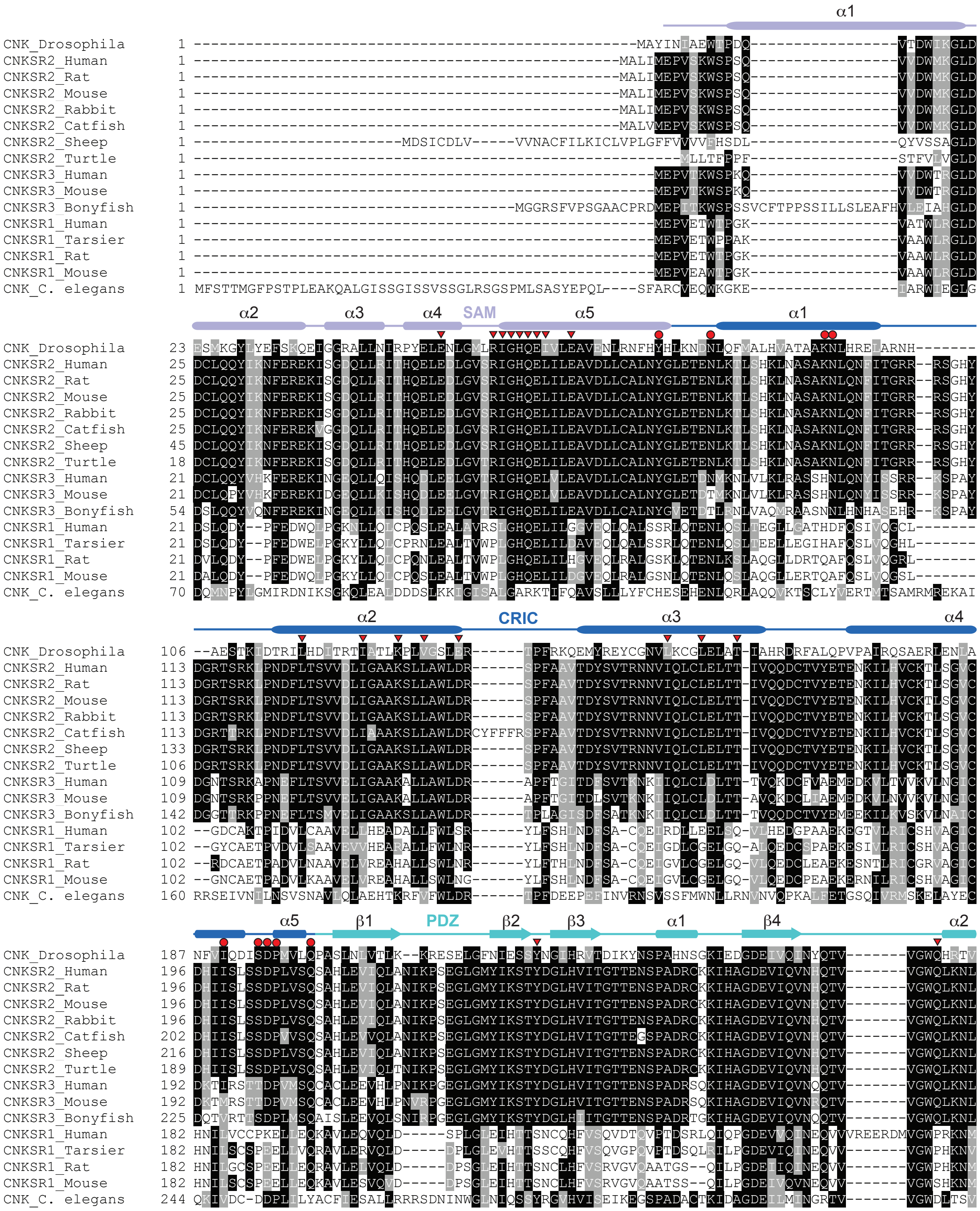

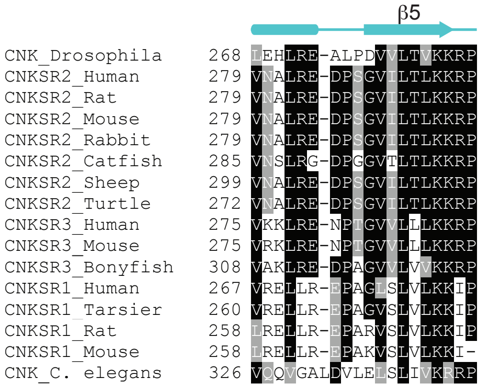

Supplementary Fig. 2 | Structure-based sequence alignment of CNK homologs.

Sequence alignment of CNK homologs from 11 metazoan species. Region encompasses only the N-terminal SAM, CRIC and PDZ domains (corresponding to the first 286 amino acids of Drosophila melanogaster CNK, top species). Secondary structure elements of the SAM. CRIC and PDZ domains are indicated on top of the alignment. Red triangles indicate residues that are conserved at the binding interface with HYP. Red circles indicate residues that are conserved at the binding surfaces with KSR and MEK. Species correspond to Drosophila melanogaster (Drosophila), Homo sapiens (Human), Rattus norvegicus (Rat), Mus musculus (Mouse), Oryctolagus cuniculus (Rabbit), Ictalurus punctatus (Catfish), Ovis aries (Sheep), Pelodiscus sinensis (Turtle), Scleropages formosus (Bonyfish), Tasrsius syrichta (Tarsier), Caenorhabditis elegans (C. elegans).

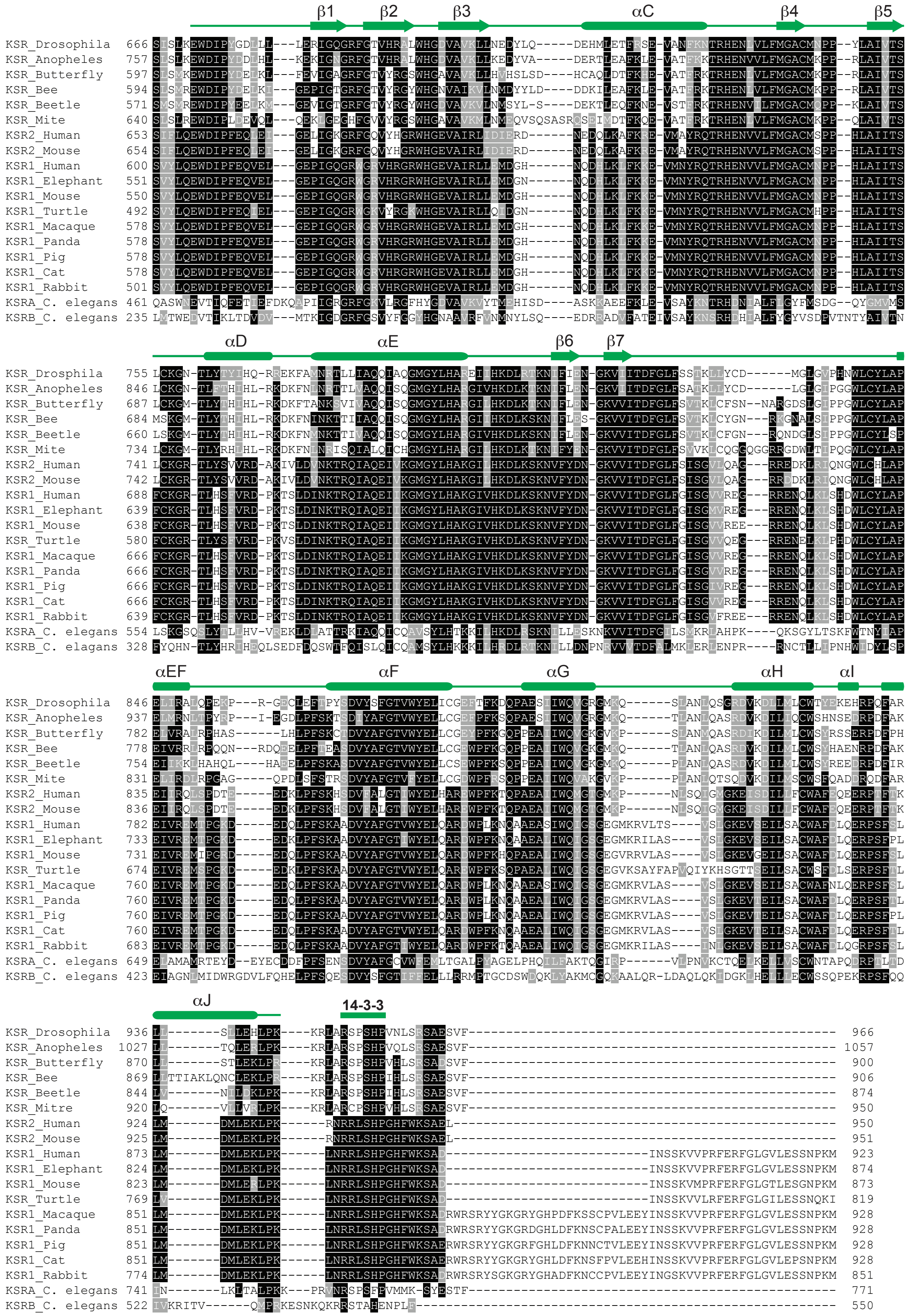

Supplementary Fig. 3 | Structure-based sequence alignment of KSR homologs.
Sequence alignment of the kinase domain of KSR homologs from 16 metazoan species. Secondary structure elements of the kinase domain are indicated on top of the alignment. Sequences encompassing the C-terminal 14-3-3 binding site consensus are highlighted in green. Species correspond to Drosophila melanogaster (Drosophila), Anopheles gambiae (Anopheles), Papilio machaon (Butterfly), Apis mellifera (Bee), Tribolium castaneum (Beetle), Tropilaelaps mercedesae (Mite) Homo sapiens (Human), Mus musculus (Mouse), Loxodonta africana (Elephant), Pelodiscus sinensis (Turtle), Macaca fascicularis (Macaque), Ailuropoda melanoleuca (Panda), Sus scrofa (Pig), Felis catus (Cat), Oryctolagus cuniculus (Rabbit), Caenorhabditis elegans (C. elegans).

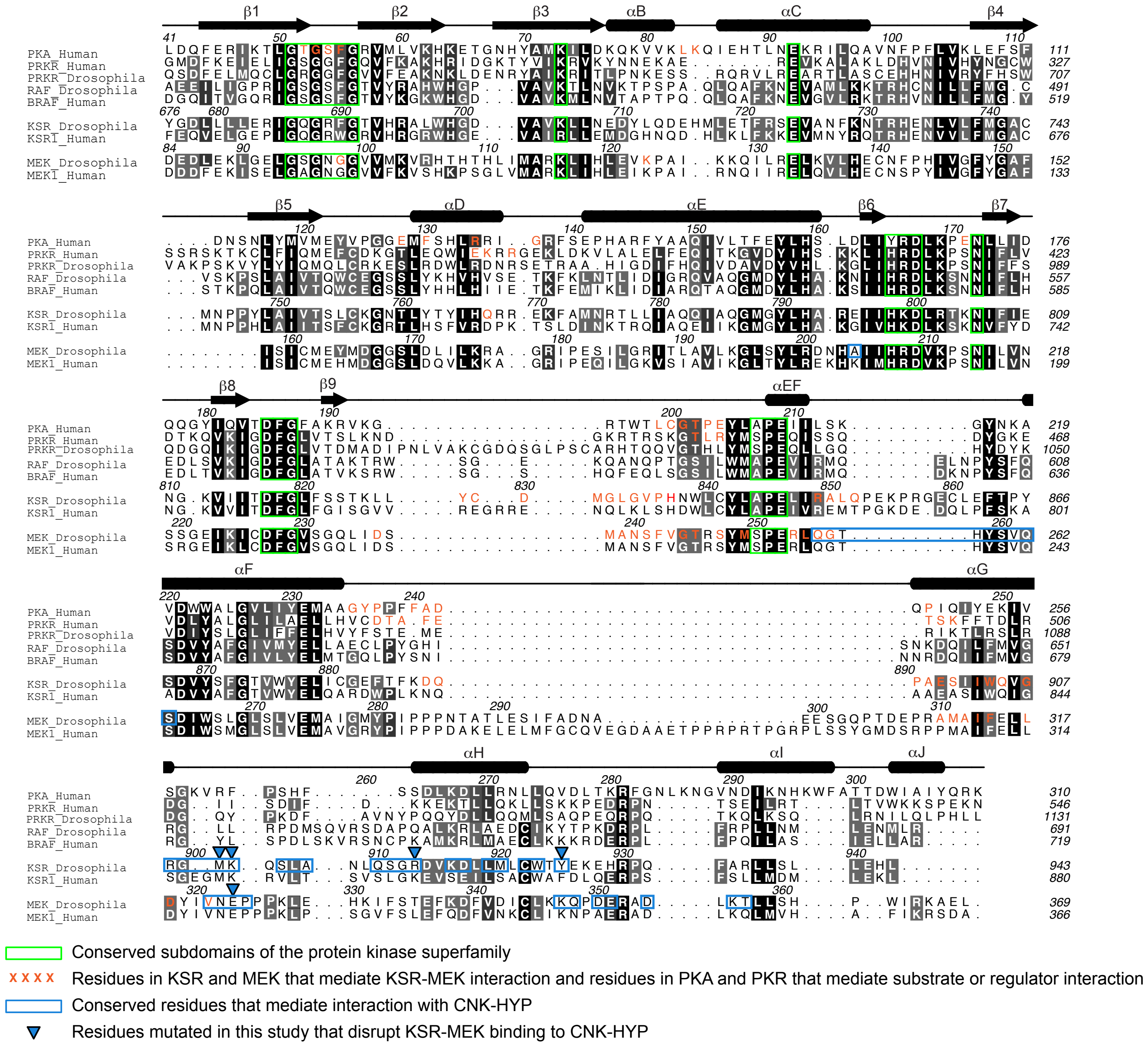

Supplementary Fig. 4 | Sequence alignment of RAF and MEK protein kinases highlighting the conserved features of the kinase fold involved in binding CNK-HYP.

Sequence alignment of the kinase domain of *Homo sapiens* (Human) PKA, *Drosophila melanogaster* (Drosophila) and *Homo sapiens* (Human) PRKR, *Drosophila melanogaster* (Drosophila) RAF, *Homo sapiens* (Human) BRAF, *Drosophila melanogaster* (Drosophila) KSR, *Homo sapiens* (Human) KSR1, *Drosophila melanogaster* (Drosophila) MEK and *Homo sapiens* (Human) MEK1. The long insert between α4 and α5 sheets of human PKR (Δ328-353) and drosophila PKR (Δ708-920) were deleted for sake of clarity. Secondary structure elements of the kinase domain of human PKA (from PDB 1ATP) are indicated on top of the alignment. Green squares highlight conserved elements of the protein kinase domain. Residues colored in orange correspond to residues located at the binding interfaces of PKA with PKI (PDB 1ATP), of PKR with eIF2α (PDB 2A1A) and of KSR with MEK (PDB 8BW9, from this study). Residues colored in light blue correspond to residues located at the binding interfaces between KSR and MEK and the CNK-HYP complex (PDB 8BW9, from this study). Residues of the kinase domain of KSR and MEK that have been shown by mutagenesis in this study to be important for binding to CNK-HYP are indicated by light blue triangles.
