# Supplementary material for: The CNK–HYP scaffolding complex promotes RAF activation by enhancing KSR–MEK interaction
Source: Nat Struct Mol Biol. 2024 Feb 22;31(7):1028–38. doi: 10.1038/s41594-024-01233-6 (PMC11257983; doi:10.1038/s41594-024-01233-6)
Supplement: Supplementary file 2 — Reporting Summary [file 41594_2024_1233_MOESM2_ESM.pdf]

nature portfolio

Corresponding author(s): Marc Therrien, Pierre Maisonneuve, Frank Sicheri

Last updated by author(s): Dec 15, 2023

Reporting Summary

Nature Portfolio wishes to improve the reproducibility of the work that we publish. This form provides structure for consistency and transparency in reporting. For further information on Nature Portfolio policies, see our Editorial Policies and the Editorial Policy Checklist.

Statistics

For all statistical analyses, confirm that the following items are present in the figure legend, table legend, main text, or Methods section.

n/a	Confirmed	
☐	☒	The exact sample size (*n*) for each experimental group/condition, given as a discrete number and unit of measurement
☐	☒	A statement on whether measurements were taken from distinct samples or whether the same sample was measured repeatedly
☐	☒	The statistical test(s) used AND whether they are one- or two-sided<br>*Only common tests should be described solely by name; describe more complex techniques in the Methods section.*
☒	☐	A description of all covariates tested
☒	☐	A description of any assumptions or corrections, such as tests of normality and adjustment for multiple comparisons
☒	☐	A full description of the statistical parameters including central tendency (e.g. means) or other basic estimates (e.g. regression coefficient) AND variation (e.g. standard deviation) or associated estimates of uncertainty (e.g. confidence intervals)
☐	☒	For null hypothesis testing, the test statistic (e.g. *F*, *t*, *r*) with confidence intervals, effect sizes, degrees of freedom and *P* value noted<br>*Give P values as exact values whenever suitable.*
☒	☐	For Bayesian analysis, information on the choice of priors and Markov chain Monte Carlo settings
☒	☐	For hierarchical and complex designs, identification of the appropriate level for tests and full reporting of outcomes
☒	☐	Estimates of effect sizes (e.g. Cohen's *d*, Pearson's *r*), indicating how they were calculated

Our web collection on statistics for biologists contains articles on many of the points above.

Software and code

Policy information about availability of computer code

Data collection	Crystallographic data were collected on the NE CAT beamline station 24-ID-C at the Advanced Photon Source (Argone, IL).<br>Cryo-EM data were collected Data on a 200 kV Talos Arctica electron microscope (Thermo Fisher Scientific), equipped with Gatan K2 summit direct electron detector (IECB, Pessac, France).<br><br>Western blot and gel band intensities were acquired using ImageJ (v. 1.54e).
Data analysis	Software, packages and pipelines used for crystallographic data analysis were the XDS package (v. 20170601), BLEND (v. 0.6.23), HKL2MAP (v. 0.4b-beta), SHELX (SHELXC v. 2016/1; SHELXD v. 2013/2; SHELXE v. 2018/2), PHASER (v. 2.8.1), COOT (v. 0.9), PHENIX (v. 1.20.1-4487), and MolProbity (v. v. 1.20.1-4487).<br><br>Software, packages and pipelines used for cryo-EM data analysis were SerialEM (v. 4.0.9), cryoSPARC (v. v3.2), Gctf (v. 1.06), UCSF Chimera (v. 1.15), UCSF ChimeraX (v. 1.6.1), COOT (v. 0.9), PHENIX (v. 1.20.1-4487) and ISOLDE (v. 1.4).<br><br>PyMol (v. 2.5.3) was used for structure visualization and figure preparation.<br><br>Statistical analysis was performed using GraphPad Prism (v. 9.5.1).

For manuscripts utilizing custom algorithms or software that are central to the research but not yet described in published literature, software must be made available to editors and reviewers. We strongly encourage code deposition in a community repository (e.g. GitHub). See the Nature Portfolio guidelines for submitting code & software for further information.

Data

Policy information about availability of data

All manuscripts must include a data availability statement. This statement should provide the following information, where applicable:

- Accession codes, unique identifiers, or web links for publicly available datasets
- A description of any restrictions on data availability
- For clinical datasets or third party data, please ensure that the statement adheres to our policy

All data supporting the findings of the current study are available within the Article and its Supplementary Information or Source Data files. Coordinates and structures for the CNK-HYP complex and for the KSR-MEK-CNK-HYP complex have been deposited in the Protein Data Bank with accession codes 8BW8 and 8BW9, respectively. The sharpened and associated maps of the KSR-MEK-CNK-HYP complex have been deposited in the Electron Microscopy Data Bank under the accession code EMD-16281.

Research involving human participants, their data, or biological material

Policy information about studies with human participants or human data. See also policy information about sex, gender (identity/presentation), and sexual orientation and race, ethnicity and racism.

Reporting on sex and gender	N/A
Reporting on race, ethnicity, or other socially relevant groupings	N/A
Population characteristics	N/A
Recruitment	N/A
Ethics oversight	N/A

Note that full information on the approval of the study protocol must also be provided in the manuscript.

Field-specific reporting

Please select the one below that is the best fit for your research. If you are not sure, read the appropriate sections before making your selection.

☒ Life sciences ☐ Behavioural & social sciences ☐ Ecological, evolutionary & environmental sciences

For a reference copy of the document with all sections, see nature.com/documents/nr-reporting-summary-flat.pdf

Life sciences study design

All studies must disclose on these points even when the disclosure is negative.

Sample size	No statistical method was used to determine sample size. A sample size of at least n=3 was used for most experiments according to standard practice for data validation and reproducibility. Biological replicates conducted on three separate days ensured data robustness.
Data exclusions	No data was excluded.
Replication	Reproducibility was demonstrated by repeating each functional and interaction studies at least three times.
Randomization	This is not relevant to our study as protein interaction experiments (co-IP, western blot analysis), crystallography and cryo-EM structure determination do not require randomization.
Blinding	No blinding was required since randomized group allocation was not performed in this study.

Reporting for specific materials, systems and methods

We require information from authors about some types of materials, experimental systems and methods used in many studies. Here, indicate whether each material, system or method listed is relevant to your study. If you are not sure if a list item applies to your research, read the appropriate section before selecting a response.

Materials & experimental systems

n/a	Involved in the study
☐	☒ Antibodies
☐	☒ Eukaryotic cell lines
☒	☐ Palaeontology and archaeology
☒	☐ Animals and other organisms
☒	☐ Clinical data
☒	☐ Dual use research of concern
☒	☐ Plants

Methods

n/a	Involved in the study
☒	☐ ChIP-seq
☒	☐ Flow cytometry
☒	☐ MRI-based neuroimaging

Antibodies

Antibodies used

Rabbit polyclonal anti-GST (1:1000; Cell Signaling, cat. number 2622)
Mouse monoclonal anti-Flag, clone M2 (1:5000; Millipore Sigma-Aldrich, cat. number F3165)
Mouse monoclonal anti-V5, clone SV5-Pk1 (1:5000; Invitrogen, cat. number 46-0705)
Rabbit polyclonal anti-MEK1/2 (1:1000 Cell Signaling Technology, cat. number 9122)
Rabbit polyclonal anti-pMEK1/2 S217/221 (1:1000; Cell Signaling Technology, cat. number 9121)
Rabbit monoclonal p44/42 MAPK, clone 137F5 (1:1000; Cell Signaling, cat. number 4695)
Mouse monoclonal anti-pMAPK, clone MAPK-YT (1:2000; Millipore Sigma-Aldrich, cat. number M8159)
Mouse monoclonal anti-CNK, clone 26A6A2 (1:10), anti-Ha, clone 12CA5 (1:200), anti-Myc, clone 9E10 (1:5), and anti-Pyo (1:5) antibodies were derived from hybridoma supernatants produced in the Therrien laboratory.

Validation

Anti-MEK, anti-pMEK, anti-pMAPK, and anti-CNK were validated using RNA interference against their respective endogenous targets in S2 cells. Anti-GST, anti-Flag, anti-Ha, anti-Myc and anti-Pyo were validated using epitope-tagged proteins.

Eukaryotic cell lines

Policy information about cell lines and Sex and Gender in Research

Cell line source(s)

Drosophila S2 cells were obtained from the Drosophila Genomics Resource Center (DGRC).
Sf9 cells were obtained from Thermo Fisher (cat. number 11496015).

Authentication

The S2 and Sf9 cells were not authenticated.

Mycoplasma contamination

The S2 cell line was regularly tested for mycoplasma.
The Sf9 cells were not tested for mycoplasma, but a fresh aliquot was used every 2-3 months.

Commonly misidentified lines
(See ICLAC register)

S2 and Sf9 cells were not listed in the ICLAC database.

Plants

Seed stocks

Report on the source of all seed stocks or other plant material used. If applicable, state the seed stock centre and catalogue number. If plant specimens were collected from the field, describe the collection location, date and sampling procedures.

Novel plant genotypes

Describe the methods by which all novel plant genotypes were produced. This includes those generated by transgenic approaches, gene editing, chemical/radiation-based mutagenesis and hybridization. For transgenic lines, describe the transformation method, the number of independent lines analyzed and the generation upon which experiments were performed. For gene-edited lines, describe the editor used, the endogenous sequence targeted for editing, the targeting guide RNA sequence (if applicable) and how the editor was applied.

Authentication

Describe any authentication procedures for each seed stock used or novel genotype generated. Describe any experiments used to assess the effect of a mutation and, where applicable, how potential secondary effects (e.g. second site T-DNA insertions, mosiacism, off-target gene editing) were examined.
