# Supplementary figures and images for: The CNK–HYP scaffolding complex promotes RAF activation by enhancing KSR–MEK interaction
Source: Nat Struct Mol Biol. 2024 Feb 22;31(7):1028–38. doi: 10.1038/s41594-024-01233-6 (PMC11257983; doi:10.1038/s41594-024-01233-6)

**Fig. 1b**

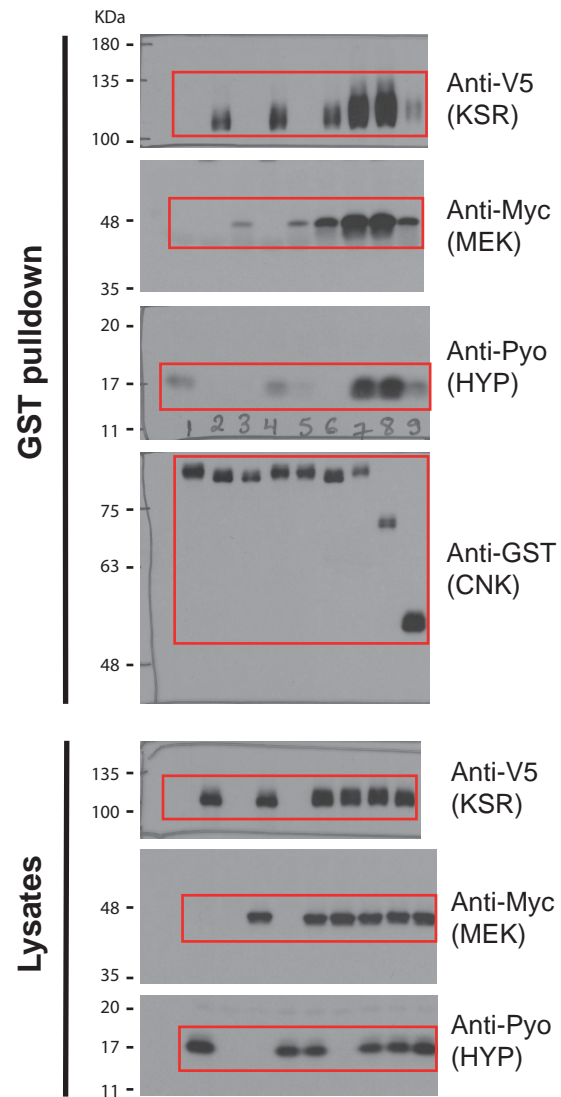

Supplement: Supplementary file 5 — Uncropped western blots. [file 41594_2024_1233_MOESM5_ESM.pdf]

**Fig. 2c**

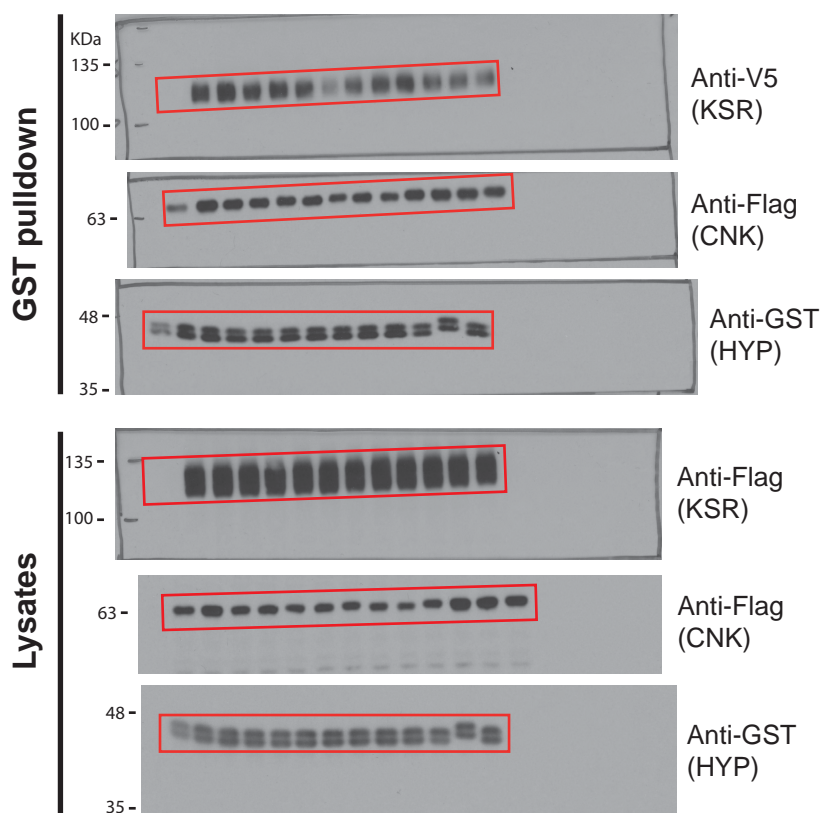

**Fig. 2d**

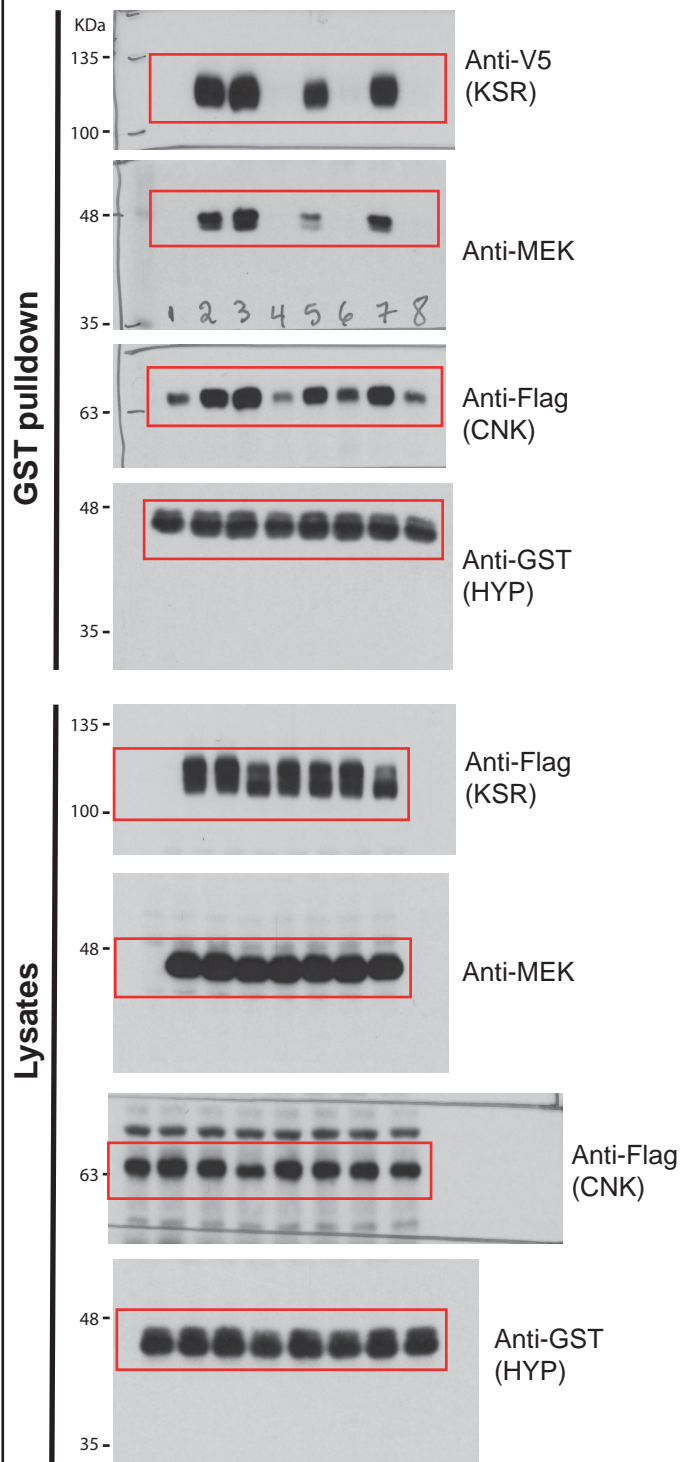

**Fig. 2e**

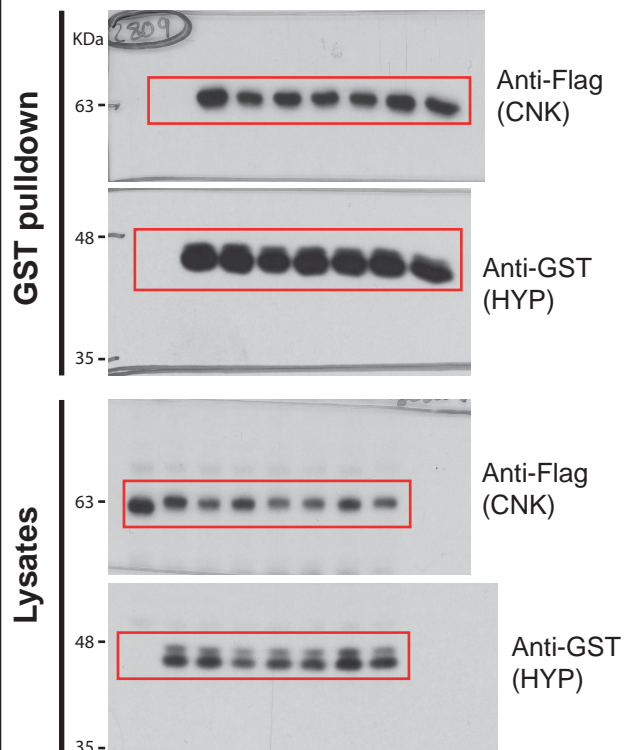

**Fig. 2g**

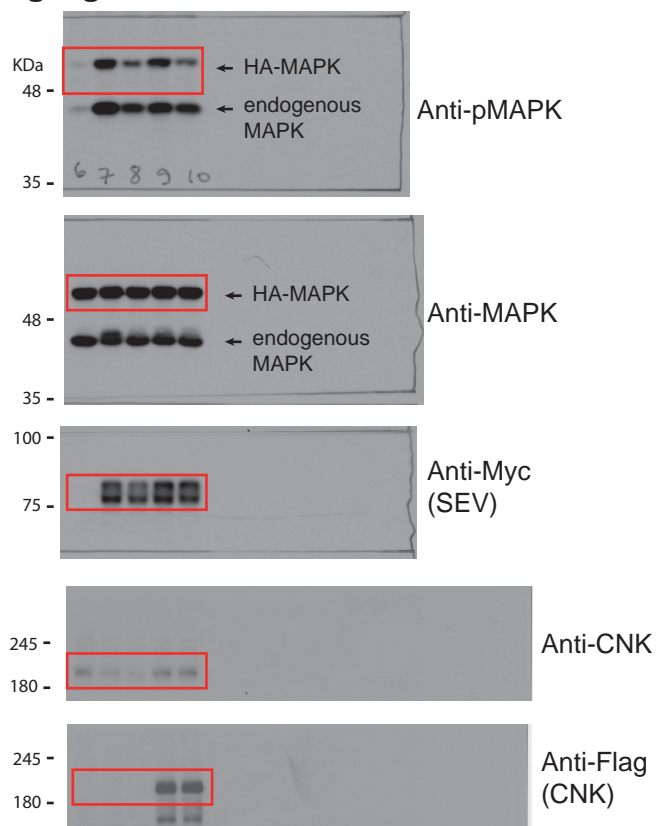

**Fig. 2f**

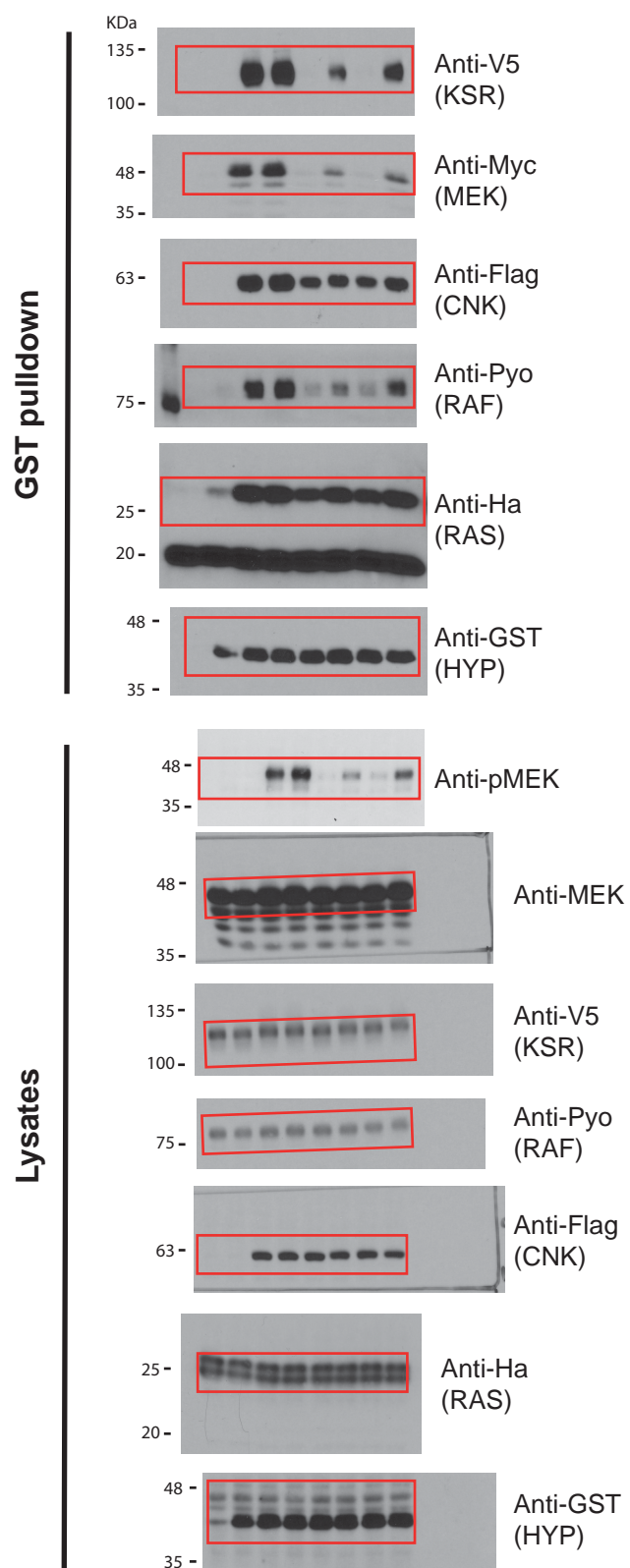

Supplement: Supplementary file 7 — Uncropped western blots. [file 41594_2024_1233_MOESM7_ESM.pdf]

**Fig. 3b**

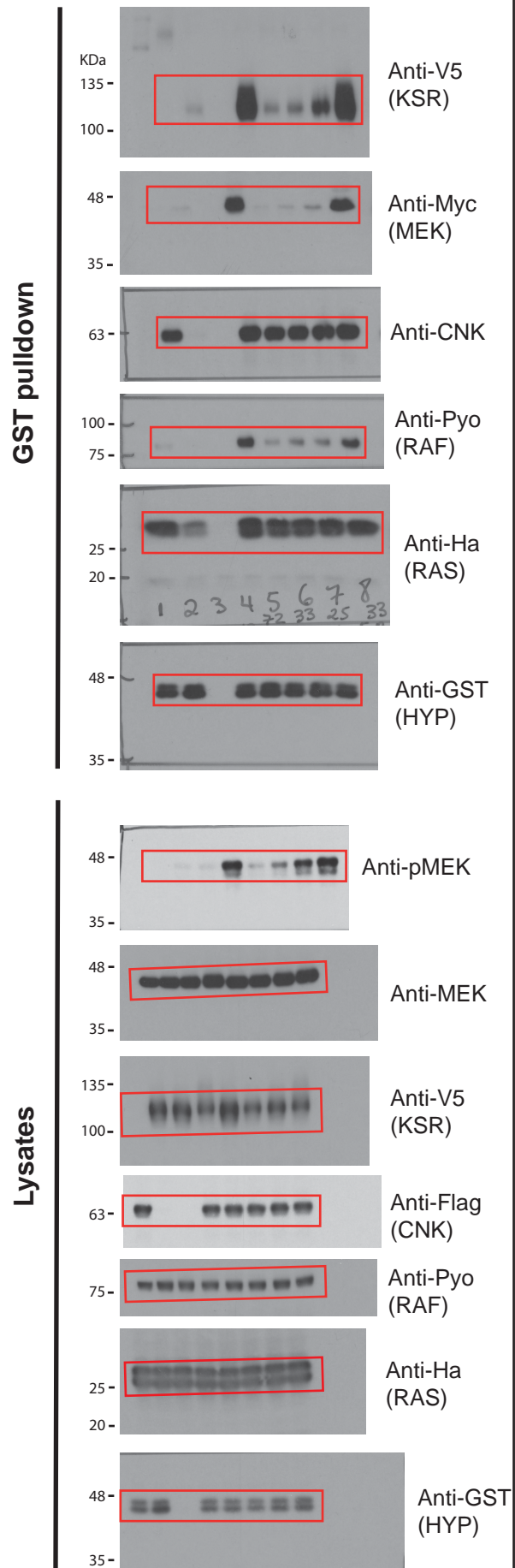

**Fig. 3c**

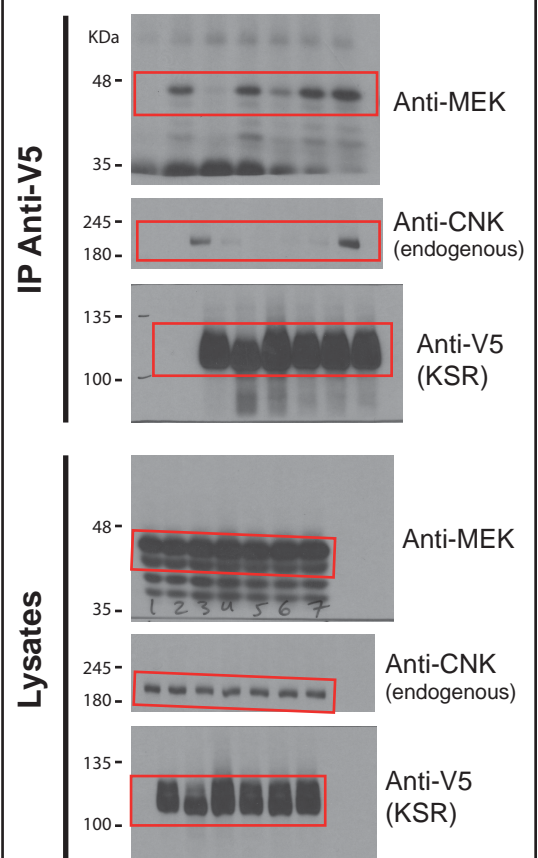

Supplement: Supplementary file 9 — Uncropped western blots. [file 41594_2024_1233_MOESM9_ESM.pdf]

**Fig. 4b**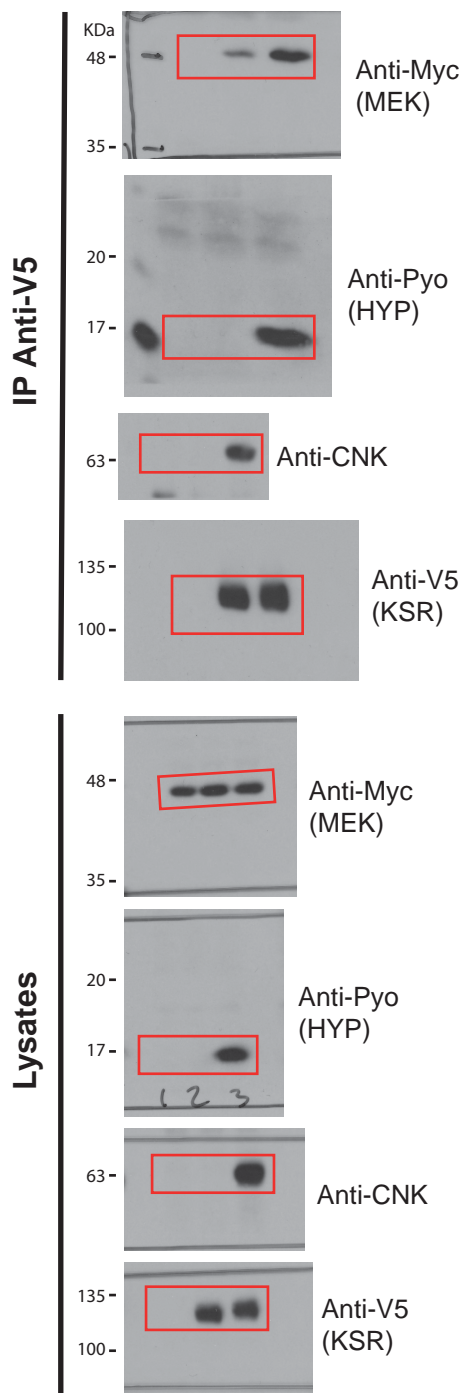**Fig. 4c**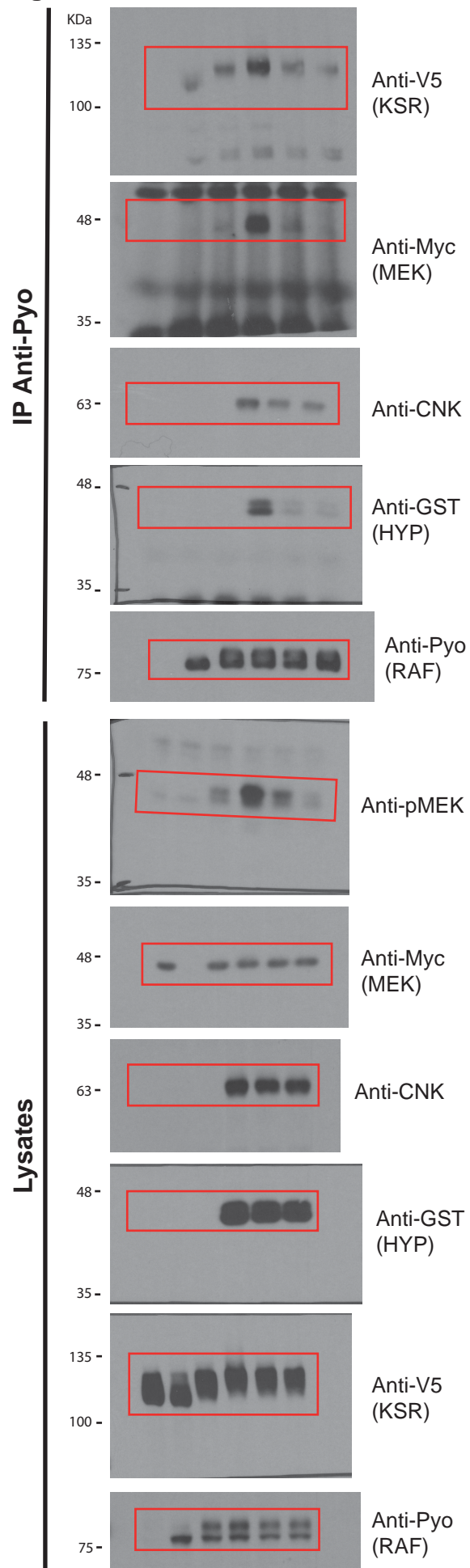

**Fig. 4d**

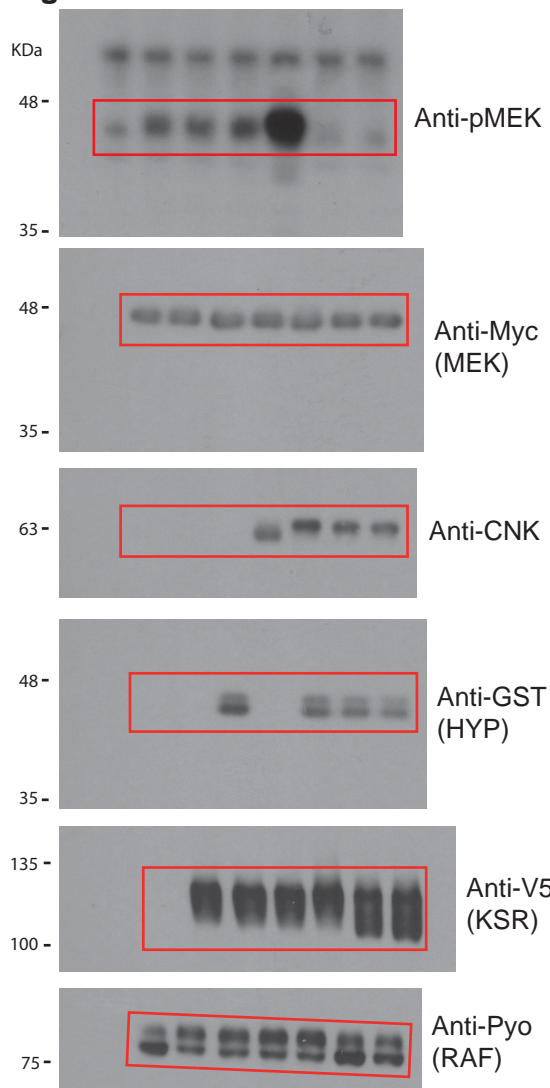

**Fig. 4e**

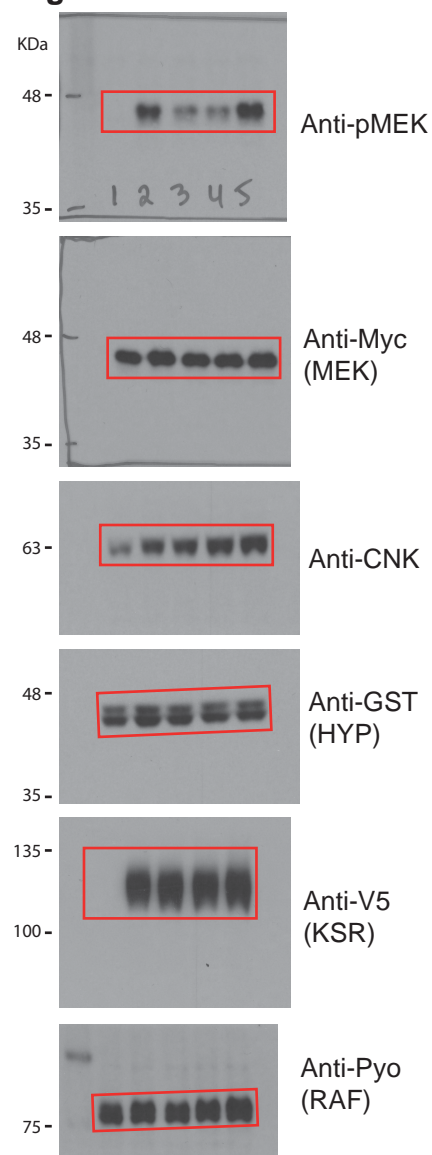

Supplement: Supplementary file 11 — Uncropped western blots. [file 41594_2024_1233_MOESM11_ESM.pdf]

Ext. Data Fig. 2b

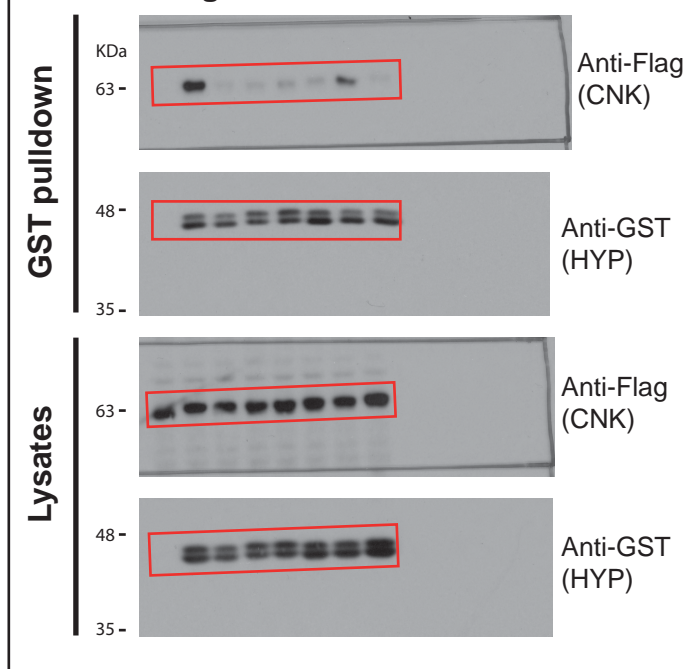

Ext. Data Fig. 2c

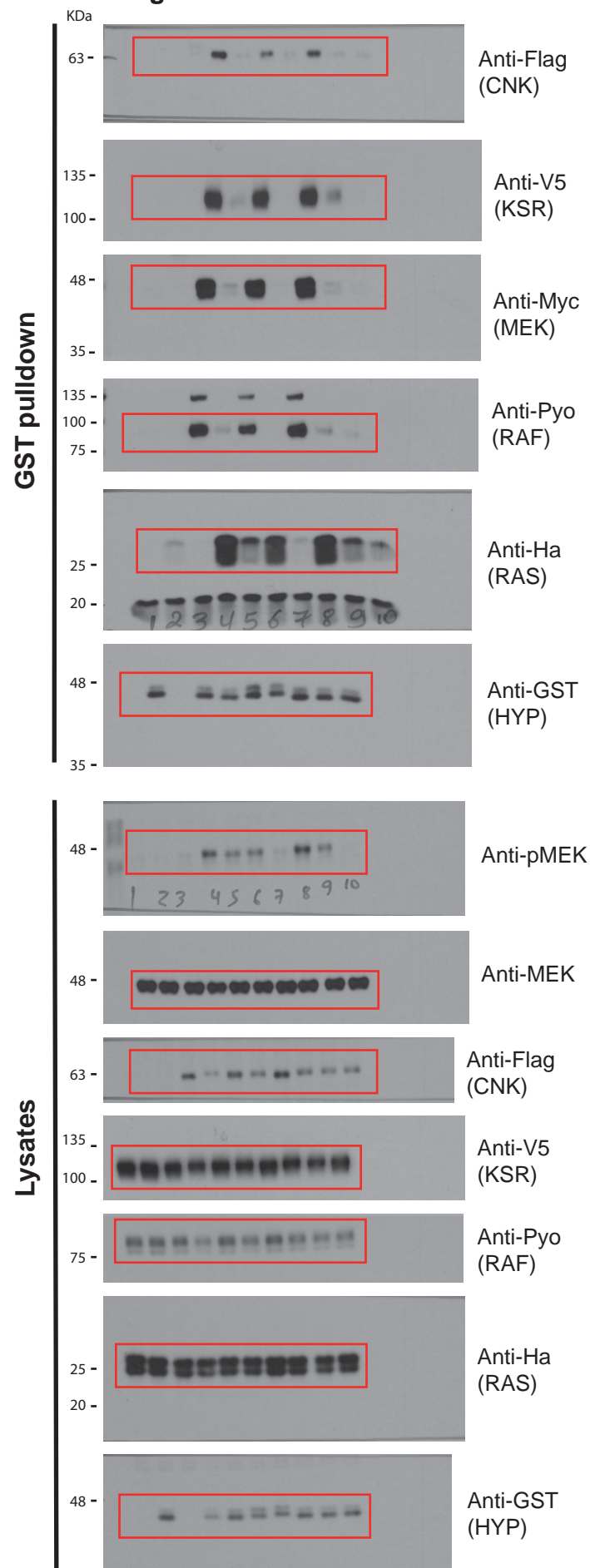

Ext. Data Fig. 2e

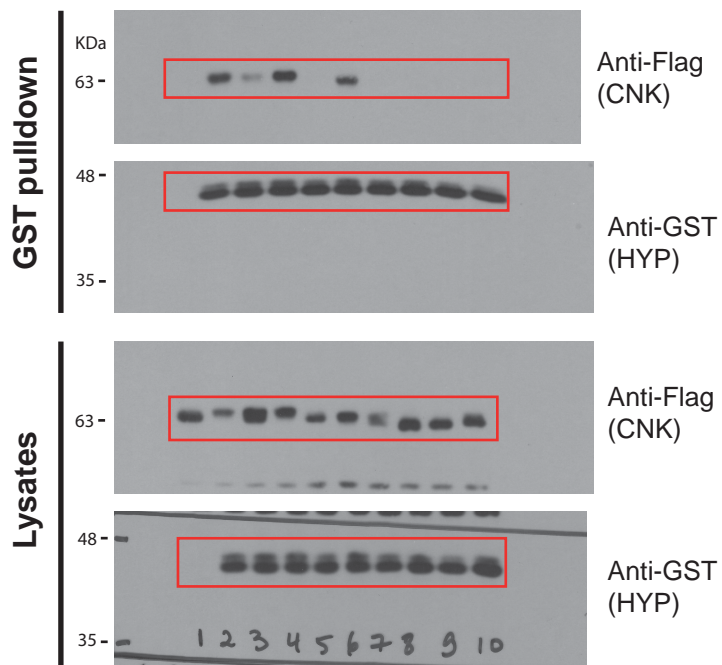

Ext. Data Fig. 2f

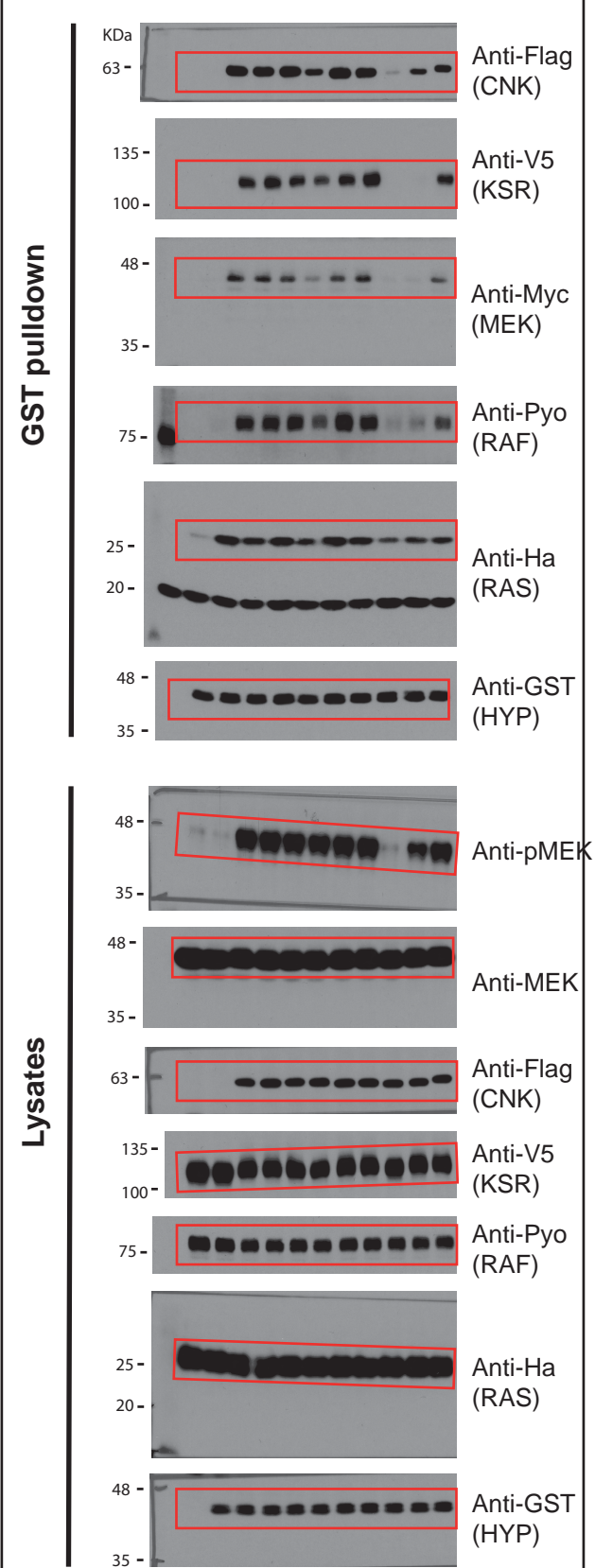

Ext. Data Fig. 2h

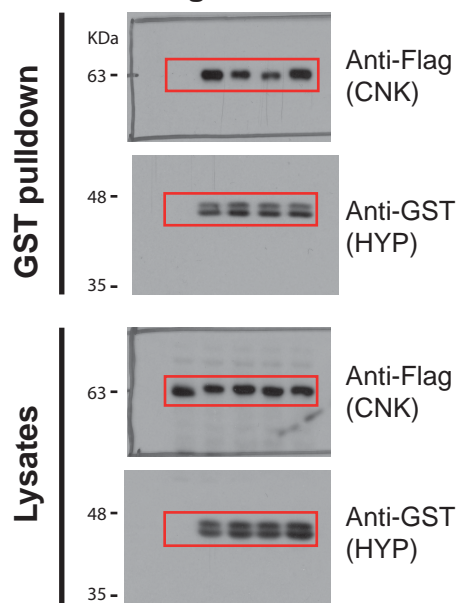

Ext. Data Fig. 2i

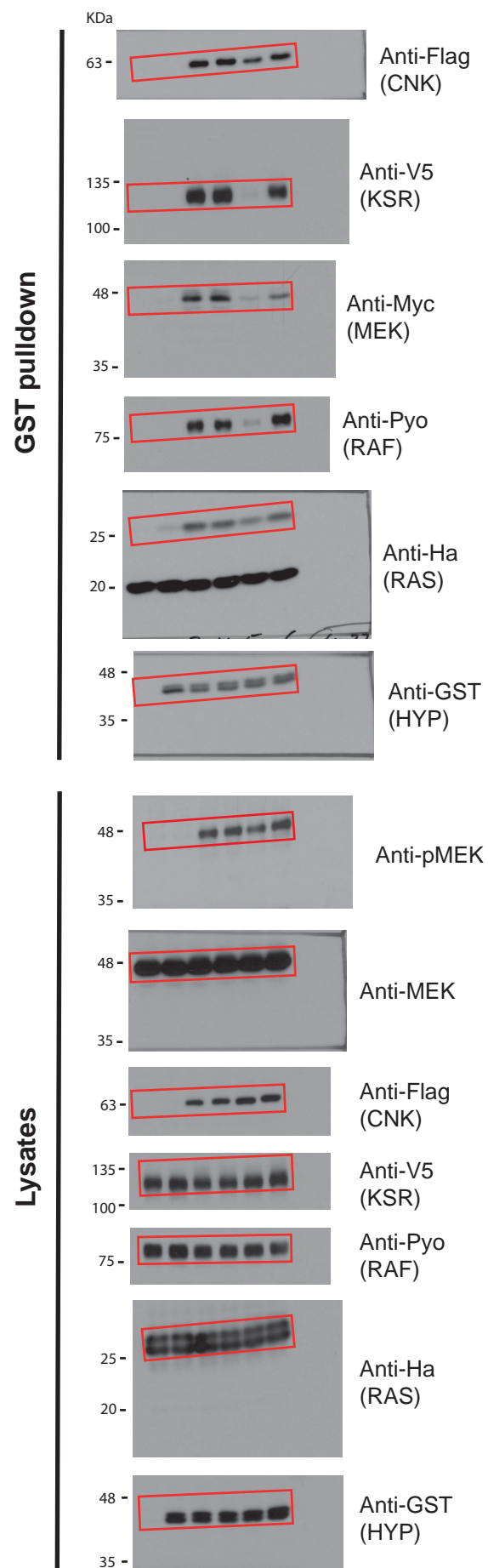

Supplement: Supplementary file 13 — Uncropped western blots. [file 41594_2024_1233_MOESM13_ESM.pdf]
