# Supplementary material for: The CNK–HYP scaffolding complex promotes RAF activation by enhancing KSR–MEK interaction
Source: Nat Struct Mol Biol. 2024 Feb 22;31(7):1028–38. doi: 10.1038/s41594-024-01233-6 (PMC11257983; doi:10.1038/s41594-024-01233-6)

Ext. Data Fig. 3a

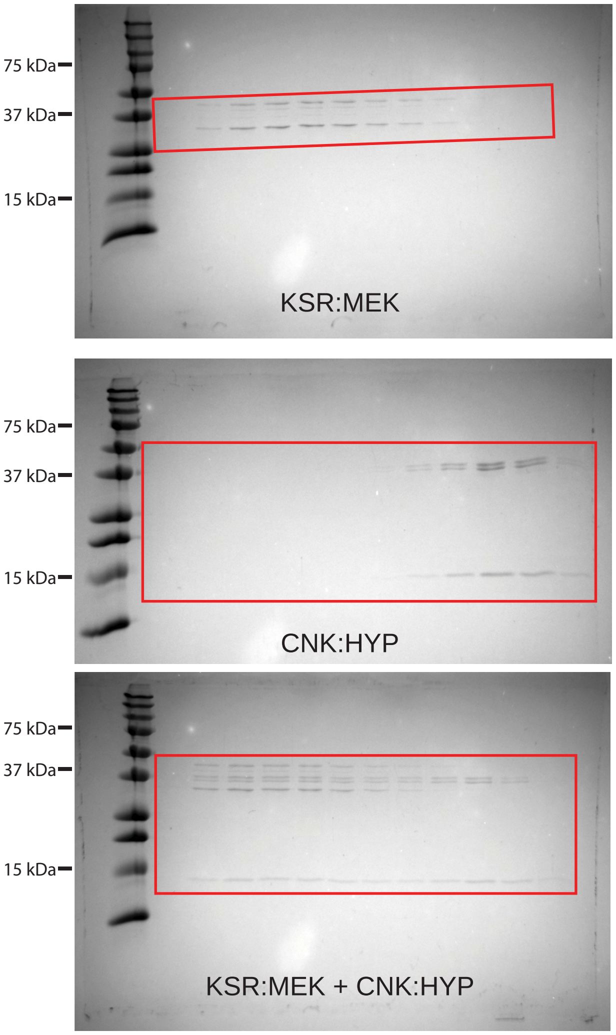

Ext. Data Fig. 3e

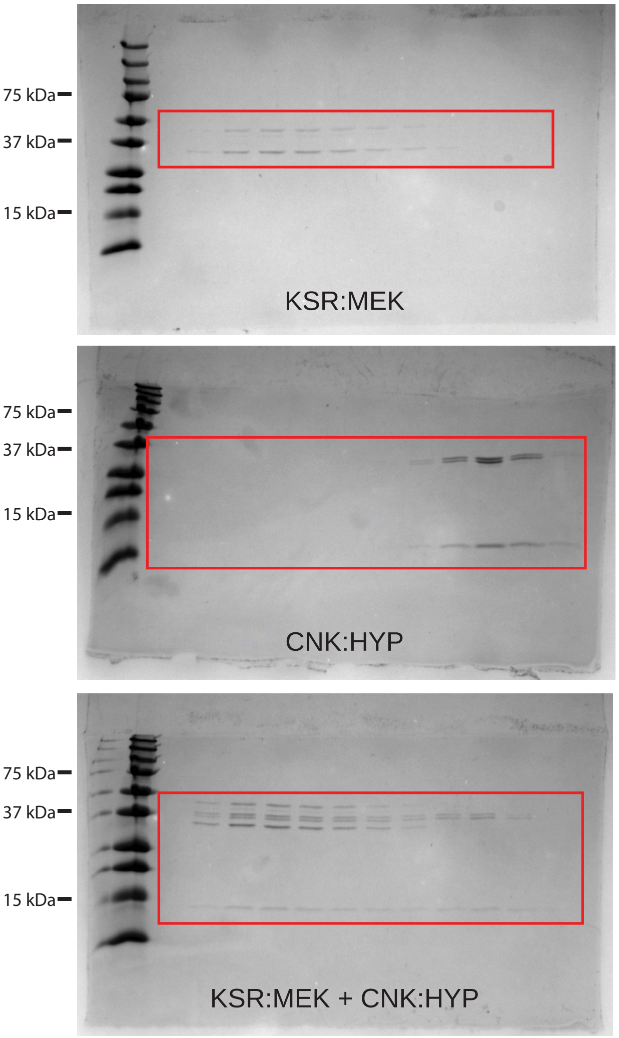

Ext. Data Fig. 3b

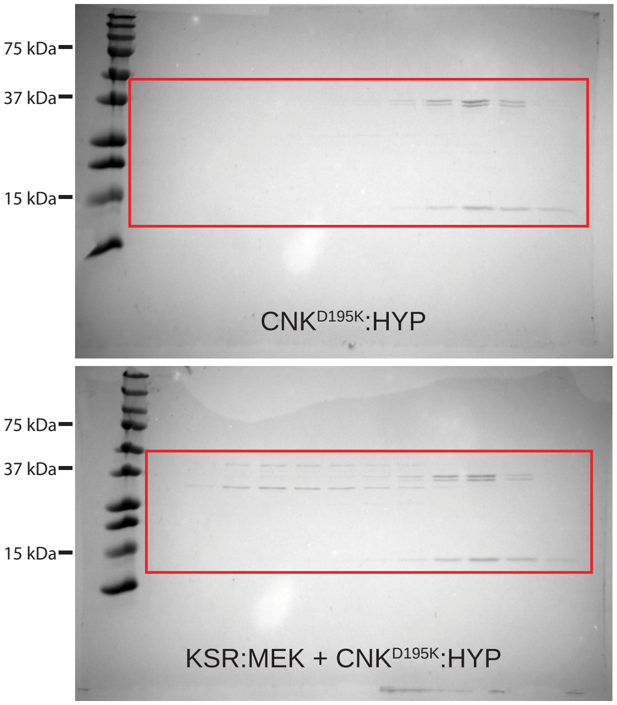

Ext. Data Fig. 3f

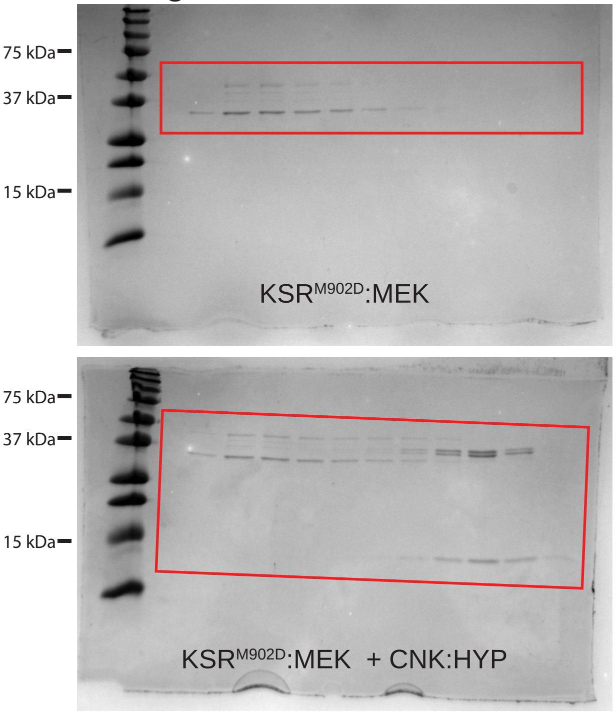

Ext. Data Fig. 3c

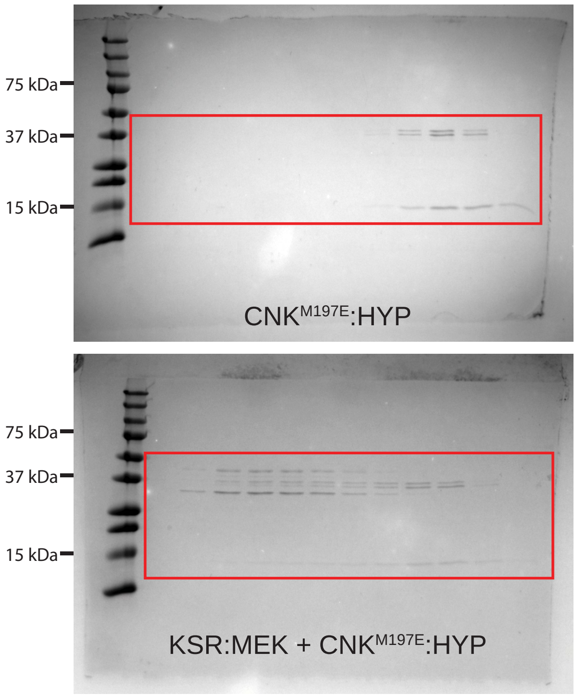

Ext. Data Fig. 3g

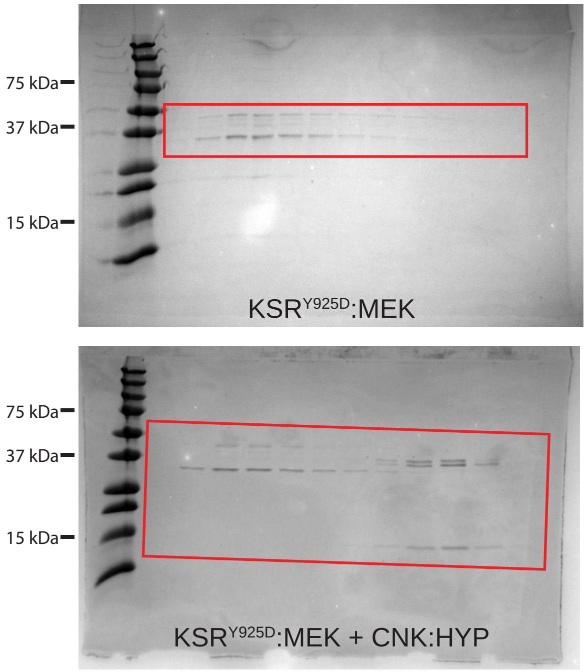

Ext. Data Fig. 3d

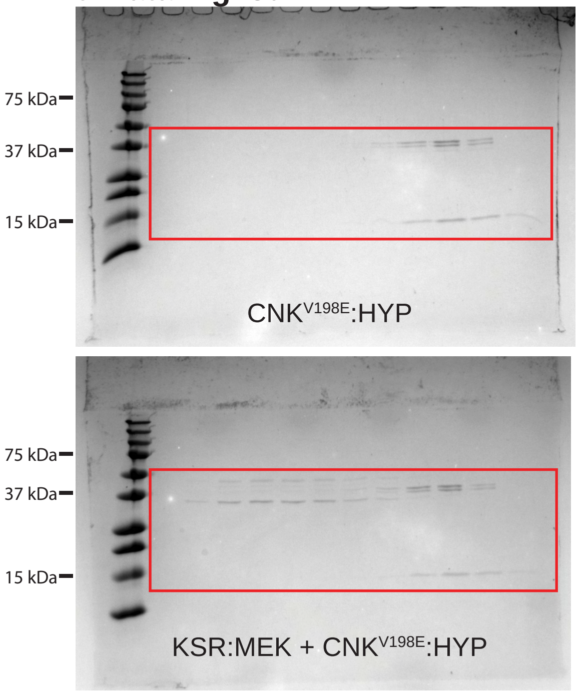

Ext. Data Fig. 3h

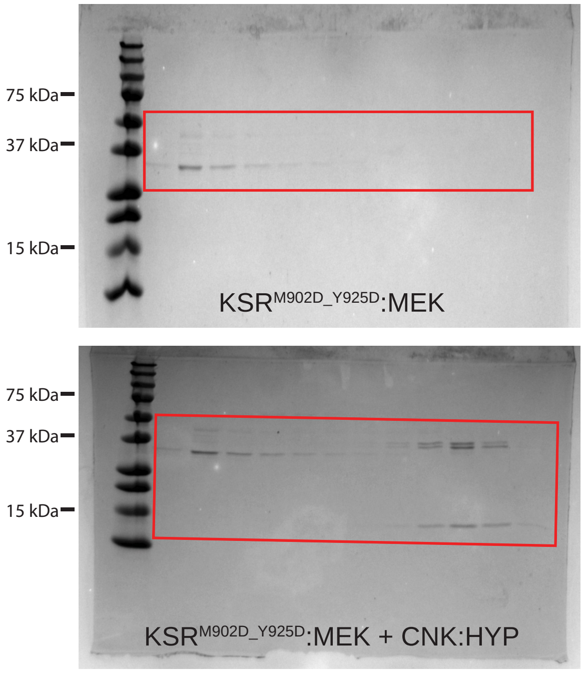

Supplement: Supplementary file 15 — Uncropped gels. [file 41594_2024_1233_MOESM15_ESM.pdf]
