# Supplementary material for: The CNK–HYP scaffolding complex promotes RAF activation by enhancing KSR–MEK interaction
Source: Nat Struct Mol Biol. 2024 Feb 22;31(7):1028–38. doi: 10.1038/s41594-024-01233-6 (PMC11257983; doi:10.1038/s41594-024-01233-6)
Supplement: Supplementary file 17 — Uncropped western blots. [file 41594_2024_1233_MOESM17_ESM.pdf]

## Ext. Data Fig. 7e

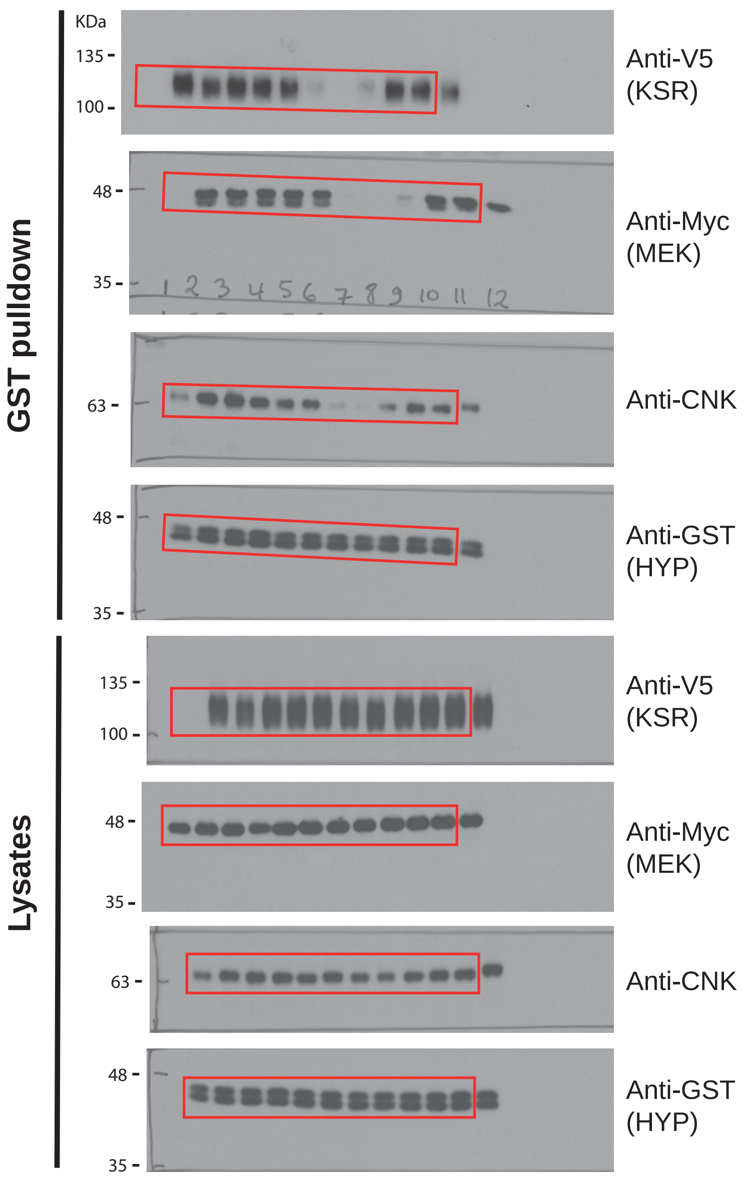


## Ext. Data Fig. 7f

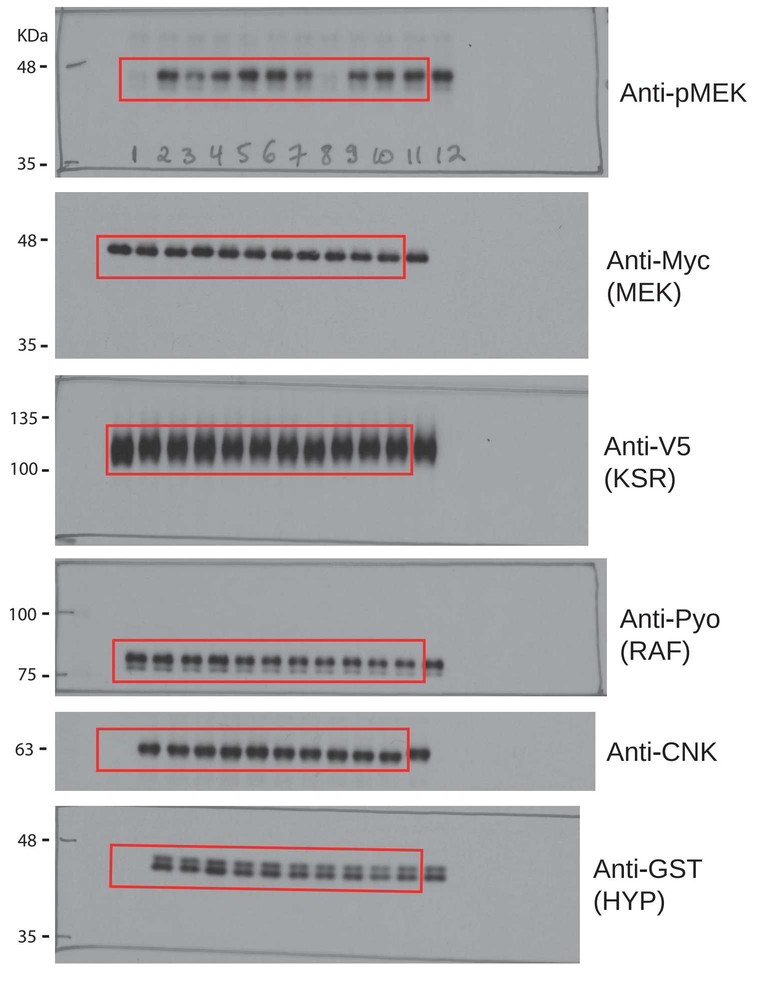

## Ext. Data Fig. 7g

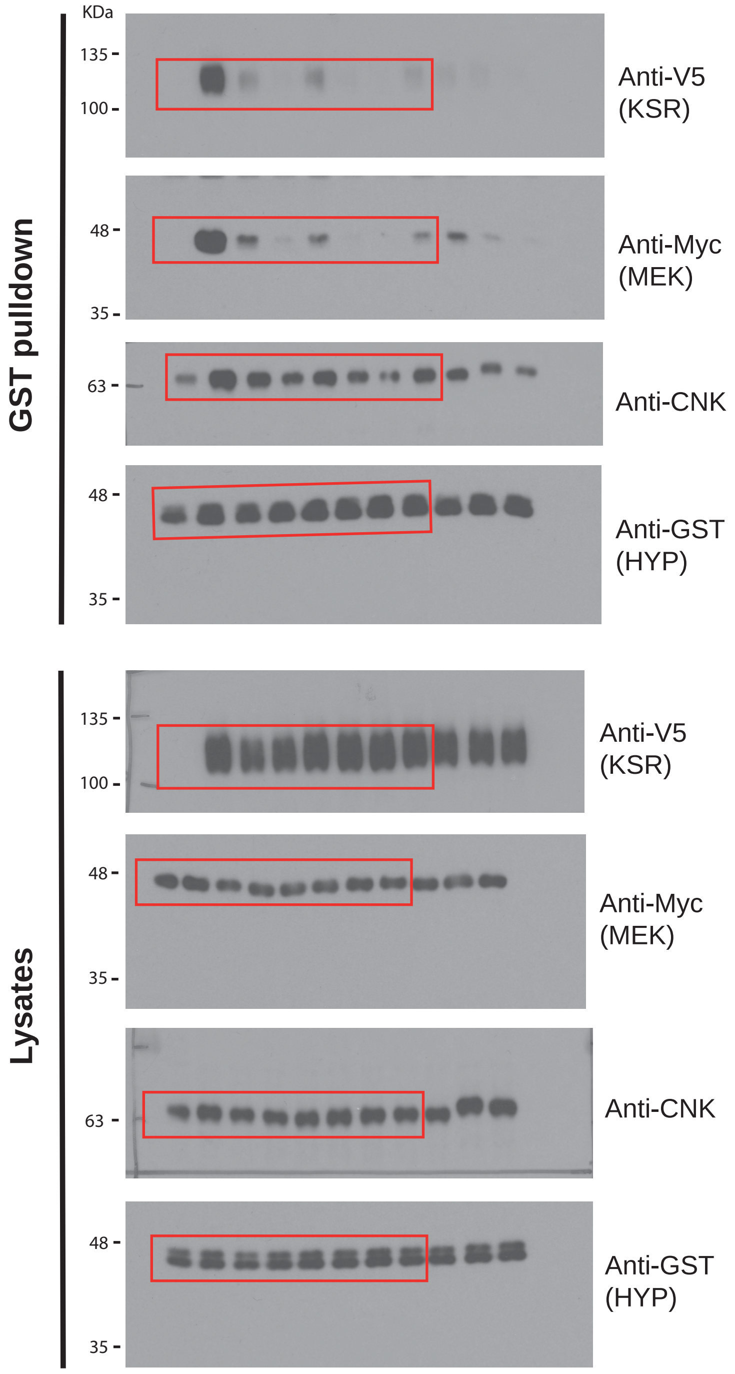


## Ext. Data Fig. 7h

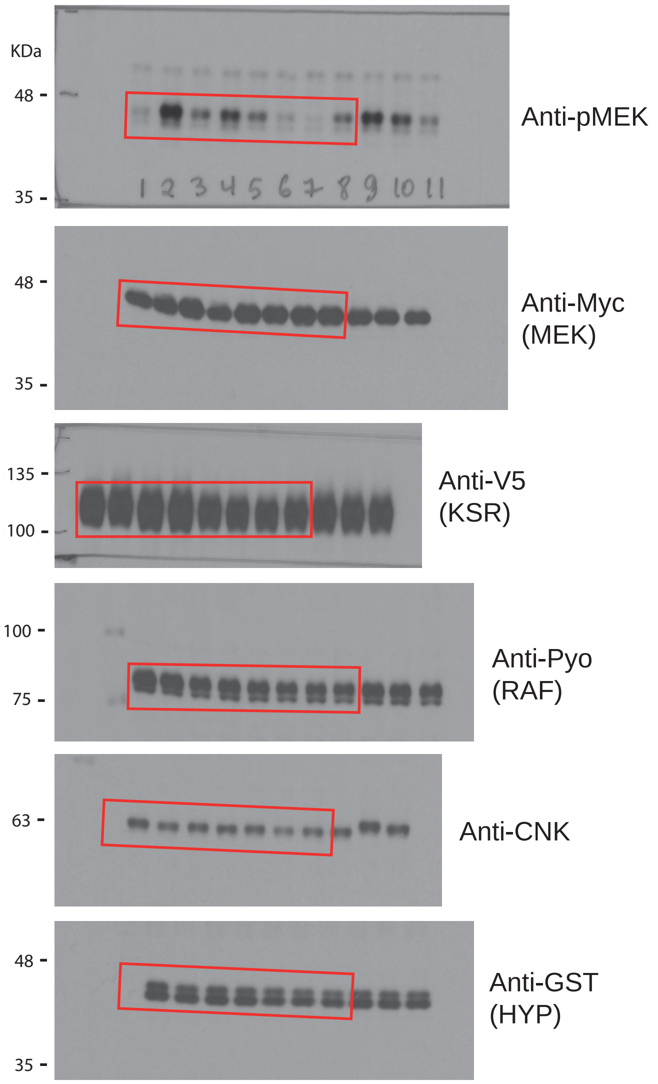

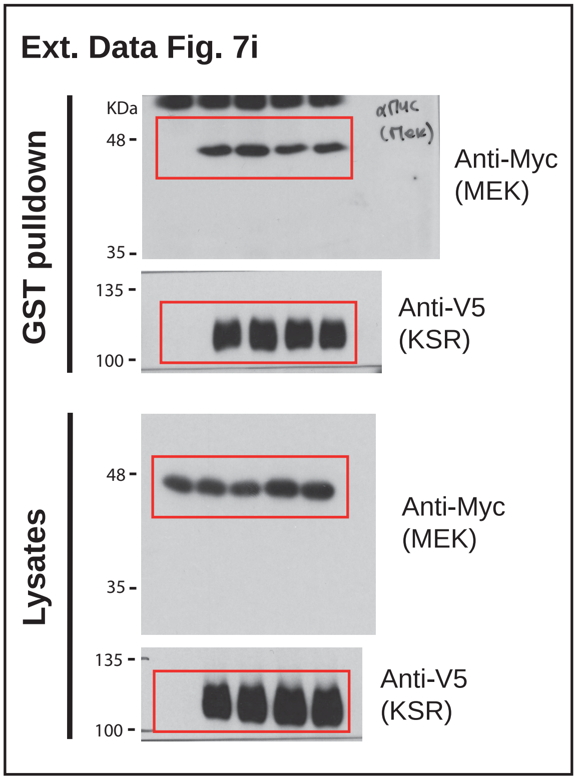
Ext. Data Fig. 7i
GST pulldown
Lysates
KDa
48
35
135
100
Anti-Myc (MEK)
Anti-V5 (KSR)

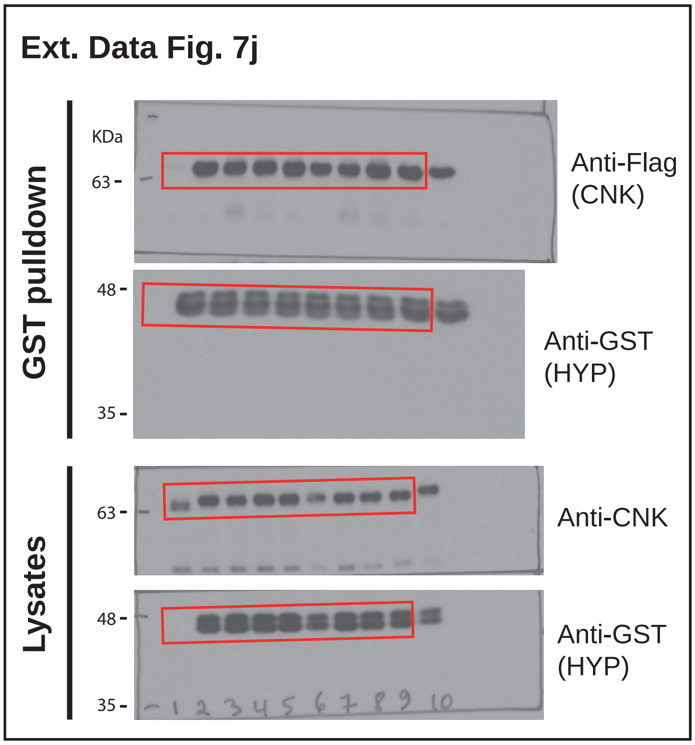
Ext. Data Fig. 7j
GST pulldown
Lysates
KDa
63
48
35
Anti-Flag (CNK)
Anti-GST (HYP)
Anti-CNK
1 2 3 4 5 6 7 8 9 10
